# Neodymium-Doped Yttrium Aluminum Perovskite (Nd:YAP) Laser in the Elimination of Endodontic Nickel-Titanium Files Fractured in Rooted Canals (Part 2: Teeth With Significant Root Curvature)

**DOI:** 10.7759/cureus.78686

**Published:** 2025-02-07

**Authors:** Amaury Namour, Marwan El Mobadder, Clément Cerfontaine, Patrick Matamba, Lucia Misoaga, Delphine Magnin, Praveen Arany, Samir Nammour

**Affiliations:** 1 Department of Dental Sciences, Faculty of Medicine, University of Liège, Liège, BEL; 2 Laser Laboratory, Department of Oral Surgery, Wrocław Medical University, Wrocław, POL; 3 Bio- and Soft Matter Division, Institute of Condensed and Nanosciences, Université Catholique de Louvain (UCL), Louvain-la-Neuve, BEL; 4 Department of Oral Biology, Biomedical Engineering and Surgery, University at Buffalo, Buffalo, USA

**Keywords:** endodontics, endodontics conservative dentistry, restorative dentistry, root canal retreatment, root canal therapy, root canal treatment complications

## Abstract

Background

In root canal treatment, fracture of nickel-titanium (Ni-Ti) instruments poses a significant challenge, particularly in teeth with complex anatomy, such as those with pronounced root curvature. A standardized and universally accepted method to address this complication remains elusive. The current study aims to assess the efficacy of neodymium-doped yttrium aluminum perovskite (Nd:YAP) laser-assisted approach in managing fractured Ni-Ti files in teeth with significant root curvature (>15 degrees).

Methods

Forty-seven fractured Ni-Ti files present in the curved portion of the root near the apex (the apical third of the root) greater than 15 degrees were included in this study. The Nd:YAP laser was applied using a power setting of 3 watts, delivering 300 mJ per pulse. A 200 µm fiber was employed, operating in pulsed mode at 10 Hertz with a pulse duration of 150 µs and an energy density of 955.41 J/cm² per second. These parameters had been previously validated for safety. The laser fiber was positioned in close proximity to the fractured file throughout the procedure. Success was defined as either complete removal or bypassing of the instrument, while failure included partial bypass, non-bypass, or lateral perforation. Scanning electron microscopy (SEM) was utilized in order to assess any physical modifications in the dentinal walls resulting from the laser irradiation. Energy-dispersive X-ray (EDX) spectroscopy was used to analyze the chemical composition of the dentinal canal walls after laser irradiation. Moreover, the average time of bypass was calculated when the bypass was possible.

Results

The overall success rate was 6.3%, in which all samples had a total bypass of the broken Ni-Ti. Of the 93.6% of failures, 74.4% experienced a lateral perforation, 10.6% a partial bypass, and 8.5% did not experience any progression or bypass. When the bypass was possible, the average time was 12.55 ± 3.26 seconds. SEM analysis revealed that the protocol results in a 26.19 µm ± 15.65 µm. EDX analysis showed that in the impact zone of irradiation, traces of 7.371 and 5.548 of Ti and Ni were shown, respectively.

Conclusions

The Nd:YAP laser-assisted protocol demonstrates low success rates in managing fractured Ni-Ti files in teeth with significant root curvature (>15 degrees). The study reveals that for future clinical experimentation, other procedures are necessary.

## Introduction

A large number of complications may possibly occur during root canal treatment and/or retreatment, particularly during the phase of cleaning and shaping of canals. Some of these complications can be effectively managed; however, others may significantly compromise treatment outcomes and, in severe cases, result in complete failure. Among these, the fracture of endodontic instruments during the shaping process is one of the most frequent and difficult challenges [1,2]. Fracture of nickel-titanium (Ni-Ti) instruments during root canal treatment is primarily caused by cyclic fatigue, torsional stress, and improper use or excessive force. Instruments made from materials such as Ni-Ti, carbon steel, and stainless steel are prone to fracture under specific conditions [3]. Several factors have been identified as contributors to instrument fracture, including the clinician’s experience, canal anatomy, tooth type, and repeated instrument usage. Additionally, rotary instruments often fail due to cyclic fatigue, while hand instruments are more prone to torsional stress. The prognosis following a Ni-Ti fracture is mainly affected by factors like the microbial contamination level, the stage of canal preparation, and, crucially, the location of the fractured instrument within the canal [4]. Hence, the prognosis depends on the location and timing, with fractures occurring after shaping and irrigation being more favorable than those at the beginning of treatment.

Various techniques are being suggested in the literature to manage fractured instruments, ranging from approaches using ultrasonic devices and hollow tubes with cyanoacrylate adhesive to microtubes and surgical approaches that involve either removing the instrument or excising the root segment containing it [5]. However, these techniques can carry considerable risks, such as perforation or a higher chance of vertical root fracture due to excessive dentin removal [5]. As there is no universally accepted protocol for handling fractured instruments in root canals, a thorough evaluation on a case-by-case basis is necessary [5,6].

Neodymium-doped yttrium aluminum perovskite (Nd:YAP) laser has emerged as a promising tool in endodontics due to its ability to cut metals with precision and efficiency [7,8]. The Nd:YAP laser is made of a crystal composed of neodymium-doped yttrium aluminum perovskite (Nd:YAlO₃). The active lasing material is neodymium ions (Nd³⁺), which are embedded in the yttrium aluminum perovskite crystal matrix. Its controlled ablation properties enable the selective removal of fractured instruments while minimizing damage to adjacent tissues. Furthermore, the laser fiber’s flexibility allows it to target the fracture site without affecting other areas of the root canal system [7]. These attributes suggest that the Nd:YAP laser could be a valuable tool for managing fractured instruments [7,8]. The current study aims to assess the effectiveness of an Nd:YAP laser-assisted protocol in removing or bypassing fractured instruments located in the apical third of teeth with significant root curvatures (>15 degrees). Building on findings from Part 1, which achieved a 100% success rate in bypassing or removing fractured instruments in teeth with minimal curvatures (<15 degrees), this study investigates whether the protocol remains effective in anatomically complex cases with root curvatures exceeding 15 degrees [9]. The null hypothesis was that the Nd:YAP laser protocol would not be successful in managing fractured instruments in teeth with significant root curvature (greater than 15 degrees).

## Materials and methods

Study design

This study aims to evaluate the efficacy of a protocol involving the use of an Nd:YAP laser. (λ: 1,340 nm, Lobel Medical, Les Roches-de-Condrieu, France) for successful retrieval of the broken/fractured instruments in root canals of teeth with significant root curvature (>15 degrees). The results of each experiment were assessed and categorized as either successful or unsuccessful. A successful outcome was determined by the complete removal of the broken instrument or successfully bypassing the fractured file. Unsuccessful outcomes included partial bypassing, lateral perforation, or failure to bypass or make progress. The ethical committee approval from the University of Liège was not required, as no human subjects were involved. The included extracted teeth were sourced for reasons unrelated to the research, and participants gave written consent for future use of their extracted teeth for research purposes.

Inclusion and exclusion criteria

This study included permanent teeth with a root curvature exceeding 15 degrees. Teeth were excluded if they exhibited any of the following: decay in the pulp chamber, radicular decay, prior endodontic treatment, narrow root canals, apexes requiring instruments larger than #25 files, internal resorption, or external resorption.

Tooth preparation

A total of 47 human permanent teeth (n = 47) with a significant root curvature (more than 15 degrees) were included in this study. All teeth were extracted for reasons that were not related to the research and were collected accordingly. After extraction, the specimens were carefully washed with distilled water and cleaned using a scaler with irrigation (Satelec, Acteon, Merignac, France) and then stored in a 0.1% thymol solution at 4°C to inhibit microbial growth. Endodontic preparation was performed manually using a standardized step-back serial technique with ISO K-Files 10, 15, and 20 (VDW GmbH, Munich, Germany) with irrigation using NaOCl 5% as in the standard of care of root canal treatment until the working length (WL) was reached for each root. The WL was determined for each canal before proceeding with further procedures using an apex locator.

Procedure for instrument fracture

A Reciproc R-25 file was used to deliberately induce instrument fractures within the root canal (2 mm tip of the RECIPROC® R25 File; VDW GmbH) that was notched at the apical 2 mm from its tip using a fine bur, removing approximately three-quarters of the file’s thickness to promote separation in the apical area. The files were then introduced into the root canal in continuous rotary motion until a fragment became lodged and subsequently fractured. To confirm the blockage caused by the fractured instrument, a 10 K-file (VDW GmbH) was utilized. The Nd:YAP laser fiber was then carefully positioned within the canals to maintain close contact with the fractured instrument. After each laser irradiation, the outcome - whether complete removal, bypassing, or failure - was assessed using a 10 K-file (VDW GmbH). To visually confirm the results, all teeth were sectioned after the procedure. Parallel radiographs were also taken to verify the location of the fractured instrument within the apical third and the precise positioning of the laser fiber in contact with the instrument fragment.

To simulate instrument fractures, the apical 2 mm of a Reciproc R-25 file (2 mm tip of the RECIPROC® R25 File; VDW GmbH) was notched using a fine bur to approximately three-quarters of the file’s thickness. This notching promoted separation in the apical region. The files were then inserted into the canal using continuous rotation until the instrument fractured and the fragment lodged, confirmed by a 10 K-file (VDW GmbH). Radiographs were taken to confirm both the location of the fractured instrument in the apical third of the canal and the laser fiber’s position in contact with the file.

Treatment protocol and laser irradiation parameters

The treatment protocol used in this study was based on a similar approach described in previous investigations [9,10]. This protocol involved three irradiation cycles, each lasting five seconds, with a 30-second resting time between each irradiation (3 × (5s + 30s RT)). The entire procedure was conducted with continuous irrigation using sodium hypochlorite (NaOCl) solution (5.25%), and approximately 6 mL of NaOCl was used for irrigation in each sample. The irradiation parameters were as follows: an output power of 3W, 300 mJ per pulse, an energy density of 955.41 J/cm², a frequency of 10 Hz, and a pulse duration of 150 µs. The laser fiber had a diameter of 200 µm and operated in contact mode. These parameters were introduced in our previous study and were suggested particularly for the removal of broken instruments. Prior to irradiation, the laser fiber was inserted into the root canal until it made close contact with the fractured instrument. A slight pressure was applied continuously during the irradiation to ensure constant contact between the fiber and the fractured instrument. A rubber stopper was placed on the fiber to mark the WL for each root canal. The total irradiation time was limited to 15 seconds for each experiment, with the irradiation process ceasing when the fiber reached the WL or at the end of the 15-second duration. The teeth samples were handheld during the entire procedure.

Evaluation method for protocol success

**Figure 1:**
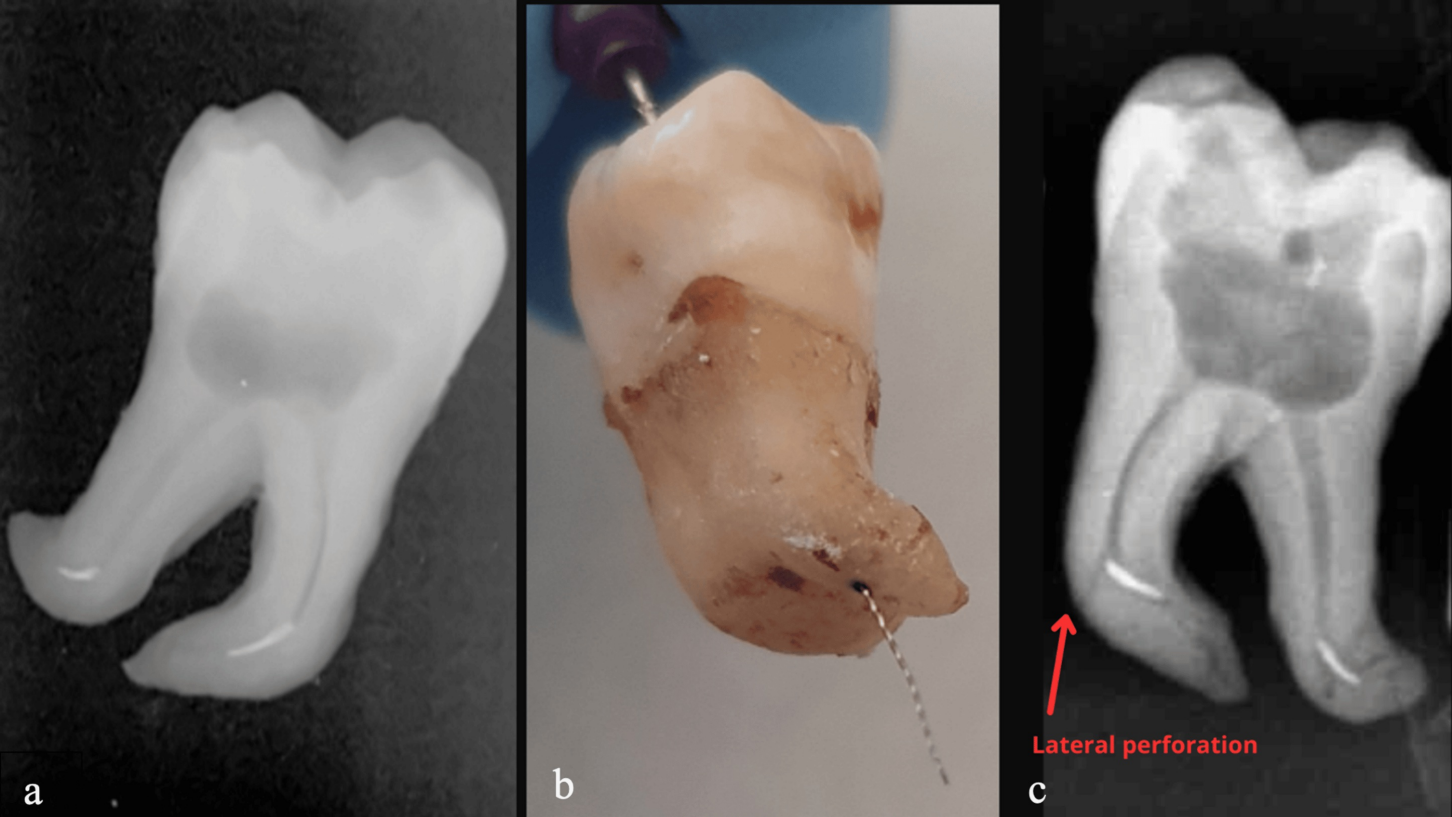
Illustration of a sample with lateral perforation obtained after the suggested treatment protocol: (a) Illustration of broken instruments inside the root canal. (b) Clinical representation of a case with lateral perforation. (c) Radiographic illustration of a lateral perforation.

At the conclusion of each irradiation cycle, a 10 K-file (VDW GmbH) was used to evaluate the results, including complete removal, bypassing, or failure of the fractured instrument. The results were classified as either success or failure based on the criteria detailed in section 2.1. To verify the outcomes, all teeth were sectioned for a second visual confirmation of the results (Figure 1).

Evaluation using scanning electron microscopy (SEM)

SEM analysis (JSM 7500F, JEOL, Tokyo, Japan) was performed to evaluate any physical changes in the dentinal walls caused by the irradiation protocol. The specimens were dehydrated using blue silica gel with a humidity indicator at room temperature, mounted onto aluminum stubs, and coated with a 25 nm gold layer through vacuum evaporation using a metallizer (model SCD 005, Bautec, Berlin, Germany). Observations were made under different magnifications, focusing on measuring the thickness of dentinal melting and the sealing of root canal tubules following laser irradiation. For each specimen, five measurements were taken to calculate the mean and SD of the dentinal layer affected by the fractured instrument’s irradiation. Statistical analysis was conducted to determine the significance of the results.

Assessment with energy-dispersive X-ray (EDX) spectroscopy

EDX spectroscopy (JSM 7500F, JEOL, Tokyo, Japan) was employed to examine the chemical composition of dentinal canal walls following laser-assisted removal of fractured instruments. This widely used technique for analyzing local chemical composition was utilized to measure the concentrations of Ni and Ti in the cervical, middle, and apical sections of each canal. Six randomly selected points in each section were assessed to determine the atomic mass percentage of Ni and Ti. The EDX peak area corresponding to each element was directly proportional to its abundance in the sample. Statistical analysis was performed to calculate the mean and SD of Ni and Ti levels for each canal section.

Statistical analysis

Statistical analysis was performed using Prism 5® software (GraphPad Software, Inc., San Diego, CA, USA) to assess the obtained mean and SD values for the time required to achieve complete removal or bypass of the fractured instrument, the thickness of the melted dentin measured with SEM, and the EDX results for each sample. A p-value of <0.05 was considered statistically significant, with a confidence level set at 99% (p < 0.001) for highly significant results. Descriptive statistics, including means and SDs, were calculated. ANOVA tests followed by a Newman-Keuls multiple comparison test (post hoc test) were used for group comparisons.

## Results

K-File bypass

Among the 47 remaining samples that underwent the treatment protocol, the overall success rate was 6.4% (n = 3). Of the 93.6% of failures (n = 44), 74.5% (n = 35) experienced a lateral perforation, 10.6% a partial bypass (n = 5), and 8.5% did not experience any progression or bypass (n = 4) (Table 1). Radiographs and root sectioning confirmed these results. Consequently, the null hypothesis was rejected.

**Table 1:** Outcomes of the protocol utilizing Nd laser for removal, bypassing, or inability to bypass the fractured instrument. Identical superscript letters indicate the absence of a statistically significant difference. Different superscripts (A, B, D) indicate the presence of a statistically significant difference. Nd, neodymium-doped

Groups	Success		Failure		
	Total elimination	Total bypass	Partial bypass	Lateral perforation	No bypass/progression
Teeth with significant root curvature (n = 47)	0%^A^	6.4%^B^	10.6%^B^	74.5%^D^	8.5%^B^

Results of SEM analysis

**Figure 2:**
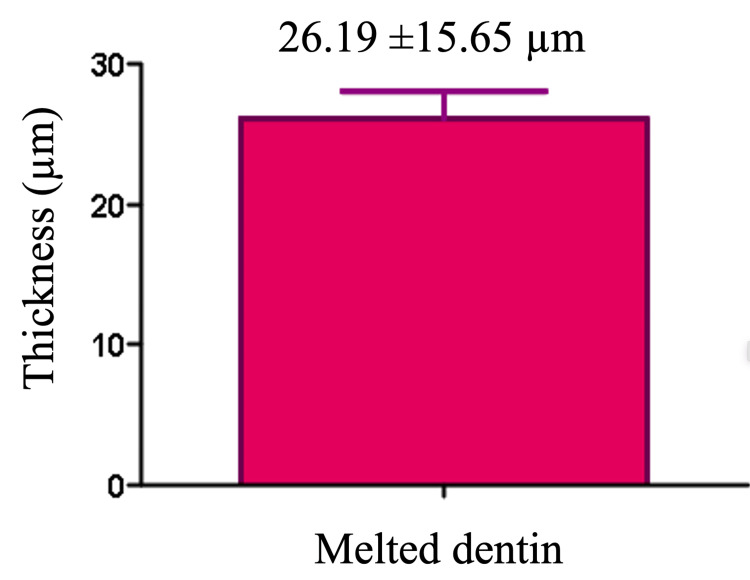
Mean value of the thickness of the melted dentin after the suggested treatment protocol with the Nd:YAP laser. Nd:YAP, neodymium-doped yttrium aluminum perovskite

**Figure 3:**
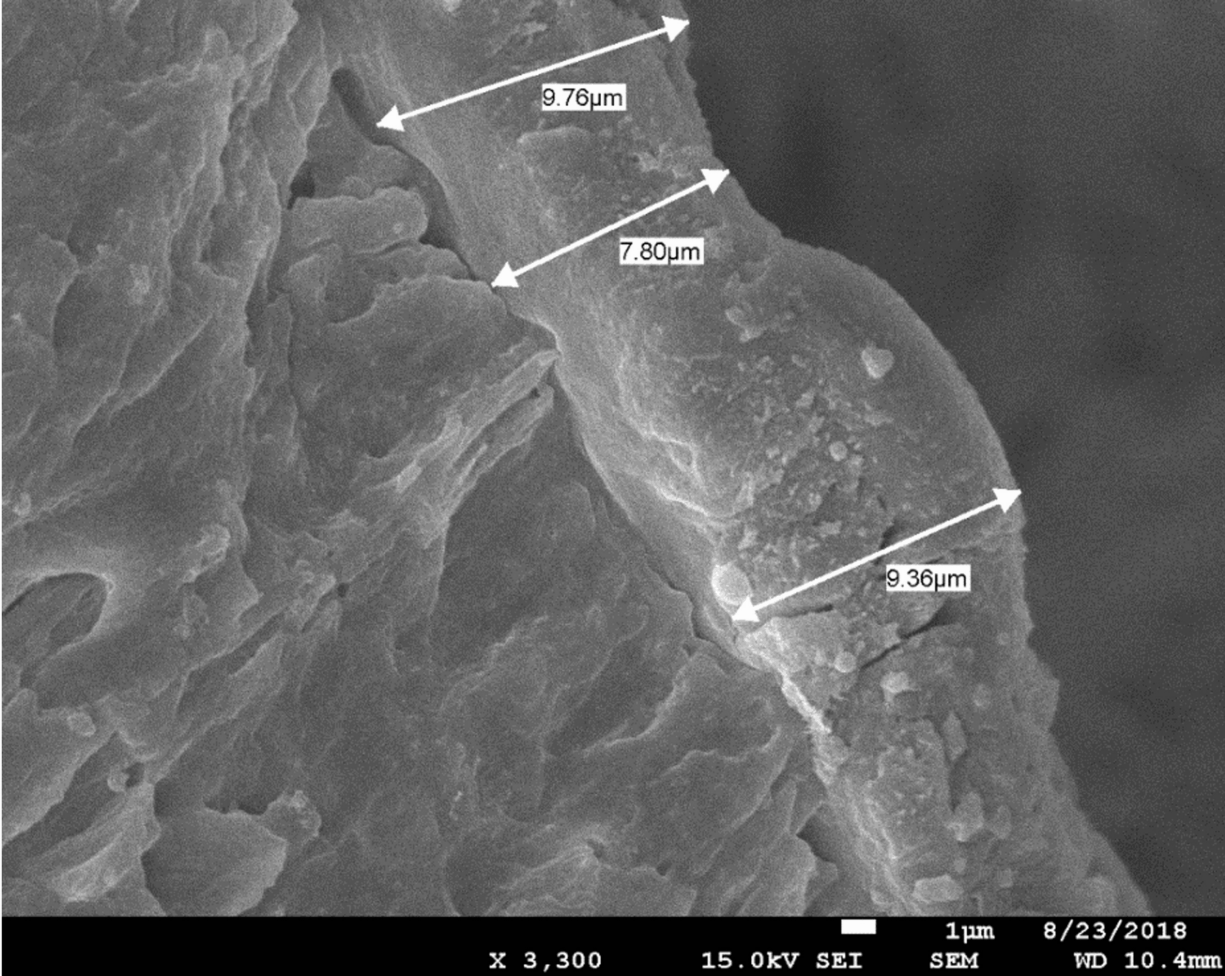
Illustration of a sample showing a clear layer of melted dentin after irradiation with the suggested Nd:YAP treatment protocol. Nd:YAP, neodymium-doped yttrium aluminum perovskite

The results obtained from SEM revealed distinct areas of melted dentin, characterized by a smooth, glass-like surface indicative of thermal alteration. Upon detailed measurement, the average thickness of the melted dentin layer was found to be 26.19 ± 15.65 µm, reflecting variability across the observed samples (Figure 2). To provide a visual representation of these findings, Figure 3 presents a cross-sectional view of one of the analyzed samples, clearly illustrating the morphological changes in the dentin caused by the thermal effects. This figure highlights the extent and uniformity of the melted layer, supporting the quantitative data.

Mean time for bypassing or removal

**Figure 4:**
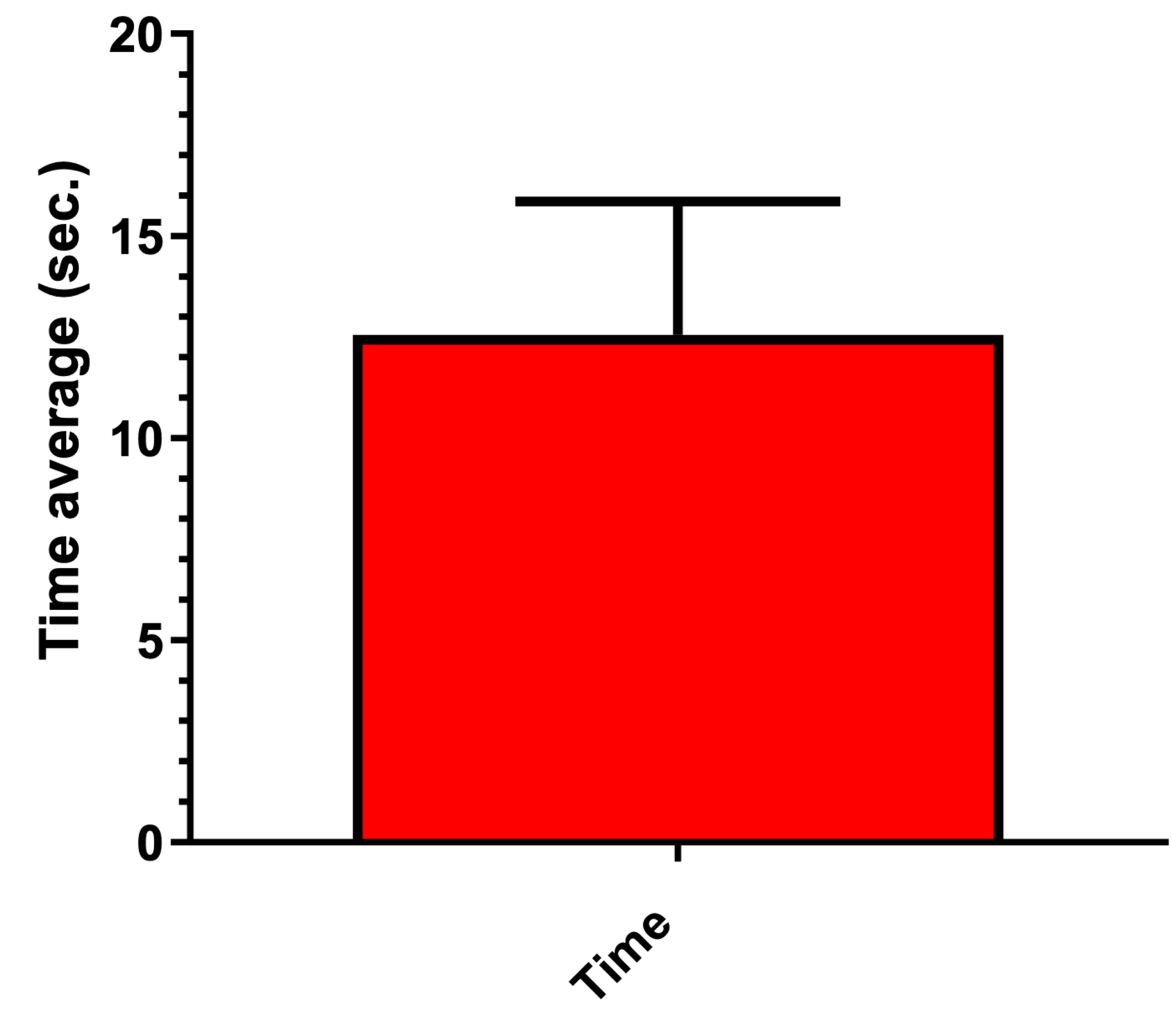
Mean time in seconds required for the complete removal and/or bypass.

When the bypass procedure was successfully performed (7%), the process proved to be relatively efficient, with an average completion time of 12.56 ± 3.29 seconds. These findings indicate that, under favorable conditions, the procedure can be carried out within a short time frame. The variation in timing reflects that, although most cases were resolved quickly, certain situations required additional time due to complexities or technical difficulties encountered during the process (Figure 4).

Results of the EDX-ray spectroscopy analysis

**Figure 5:**
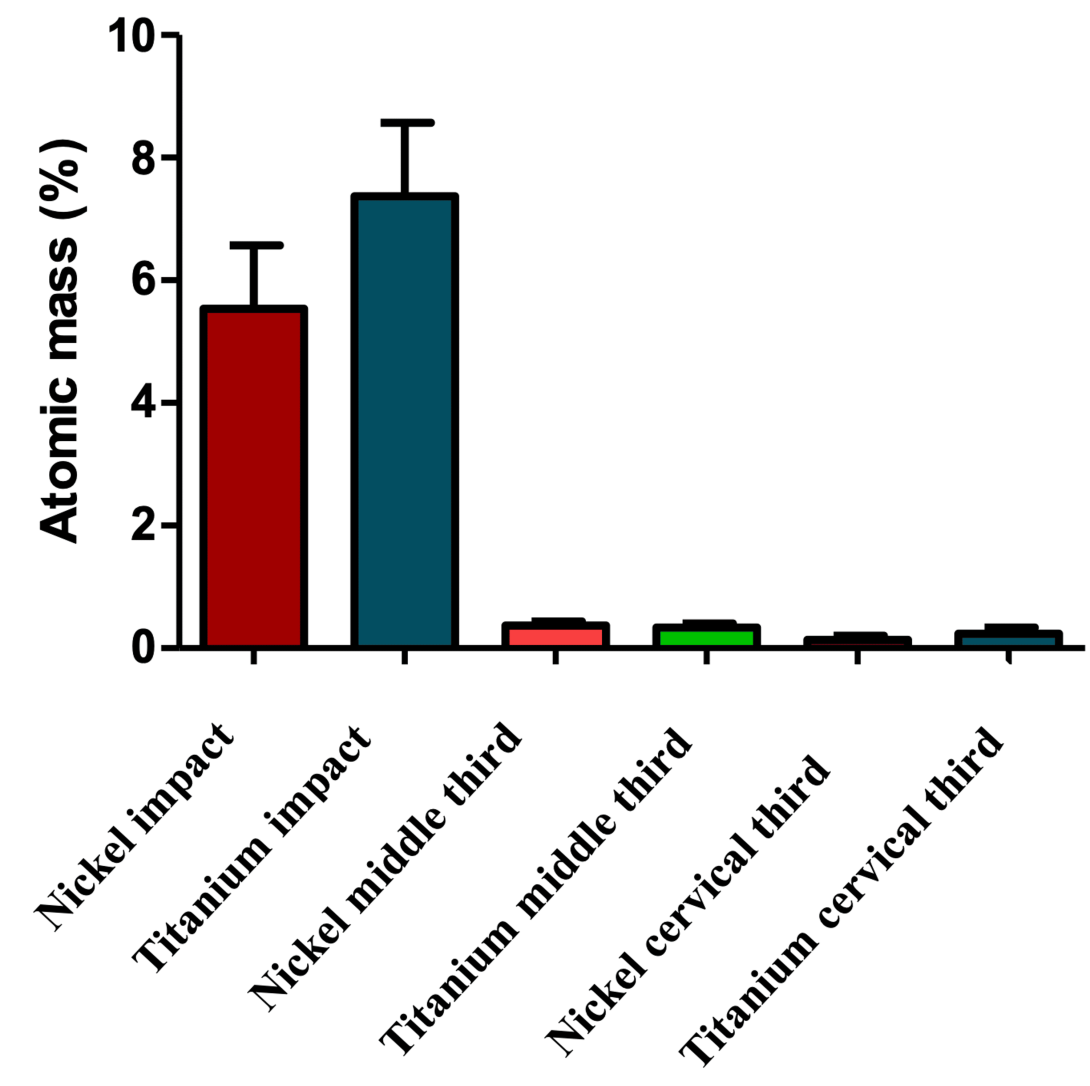
Chemical composition in Ni and Ti at different parts of dentinal canal walls. Values are expressed in atomic mass percentage (%). Ni, nickel; Ti, titanium

The mean and SD values for Ni and Ti detected on the dentinal canal walls of all studied samples were 5.54% ± 4.62 and 7.37% ± 5.39, respectively, within the irradiation area at the apical third. The presence of Ni and Ti traces decreased along the root canals to the cervical direction. At the cervical third and middle third of treated canals, the levels of Ni and Ti were 0.53% ± 0.23 and 0.31% ± 0.27, respectively, while apical to the impact area, they measured 0.13% ± 0.17 and 0.22% ± 0.26, respectively (Table 2, Figure 5).

**Table 2:** Chemical composition of dentinal walls in Ni and Ti at different parts of the canals: cervical third, middle third, and apical thirds of roots (impact zone). Values are expressed in percentage of atomic mass (%). Different superscript letters indicate statistically significant differences (A; B; C; D and a; b); similar superscript letters indicate no statistically significant difference (b; b). Ni, nickel; Ti, titanium

	Impact zone (apical third of roots)		Cervical third of roots		Middle third of roots	
	Ni	Ti	Ni	Ti	Ni	Ti
Mean	5.548^A^	7.371^a^	0.3513^B^	0.3106^b^	0.1318^C^	0.2291^b^
SD	4.621	5.393	0.23	0.2764	0.1786	0.2646

## Discussion

Instrument separation, or the fracture of endodontic instruments, is a significant complication in root canal therapy [11]. This issue can severely impact the cleaning, shaping, disinfection, and filling stages of treatment, ultimately compromising the success of endodontic procedures. Although several techniques have been proposed for managing instrument separation, no universally accepted protocol has been established. It is generally agreed that successful management involves either the complete removal or bypassing of the fractured instrument [11-13]. The present study aimed to evaluate the efficacy of an Nd:YAP laser-assisted protocol for managing fractured Ni-Ti instruments in teeth with significant root curvatures. The Nd:YAP laser was selected for its effective interaction with Ni-Ti, a material commonly used in endodontics, and its favorable absorption properties in the near-infrared spectrum. Studies have demonstrated the Nd:YAP laser’s ability to disintegrate Ni-Ti fragments while minimizing damage to surrounding tissues. However, the findings of this study indicate that while the protocol shows promise in some cases, it did not achieve a high success rate in teeth with significant root curvatures. The results revealed that only 6.38% of the cases were successful, while 93.6% failed. Among the failures, 10.63% involved partial bypasses, 74.4% resulted in lateral perforations, and 10.6% showed a complete failure to bypass the fractured instrument. These findings highlight the limitations of the Nd:YAP laser-assisted protocol in addressing the challenges posed by significant root curvatures.

Several factors contribute to the low success rate observed in this study. Primarily, significant root curvature poses a major challenge to the laser’s ability to accurately target and treat the fractured instrument. In canals with curvatures exceeding 15 degrees, the Nd:YAP laser’s capacity for precise targeting is reduced. The energy from the laser may be unevenly distributed, reducing its effectiveness in disintegrating or bypassing the fractured Ni-Ti instrument. In contrast to straight or minimally curved canals, the complex anatomy of severely curved canals exacerbates these challenges. Lateral perforations, observed in 74.4% of the failed cases, suggest that misdirected laser energy or excessive energy application caused unintended damage to the dentinal walls. Lateral perforation is a well-documented complication in curved canals and underscores the importance of careful management of laser parameters in these situations [14]. While the Nd:YAP laser has been shown to effectively disintegrate Ni-Ti fragments in straight canals, as concluded in Part 1 of this study, its performance is less reliable in the irregular geometries of curved canals [9]. Achieving the precision needed to interact with the fractured instrument without damaging the surrounding dentin is more challenging in such cases, leading to a higher incidence of lateral perforations. Despite the promising theoretical potential and previous studies supporting the use of the Nd:YAP laser in simpler canal systems, this study suggests that its application in canals with significant curvatures requires further refinement. Adjustments to the irradiation parameters and the incorporation of additional techniques may be necessary to overcome these limitations. While prior research has shown that lasers are effective in removing canal sealers and fractured instruments in straight, minimally curved canals, the complex canal anatomy encountered in this study likely limited the Nd:YAP laser’s efficacy. Importantly, this study confirmed the Nd:YAP laser’s safety in preserving dentin, as no significant dentinal damage was observed during irradiation. However, the high rate of lateral perforations emphasizes the need for improved fiber manipulation and stricter control over laser parameters in cases involving significant root curvatures.

The limitations of this study include a small sample size and focus on teeth with significant curvatures. Although the study aimed to address challenges in complex root canal systems, further research involving larger sample sizes and a broader range of canal anatomies is required. Additionally, comparing the Nd:YAP laser protocol with other laser systems or techniques in teeth with significant curvature could provide a more comprehensive evaluation of its effectiveness.

While the Nd:YAP laser shows potential for managing fractured instruments in root canals, its application in teeth with significant curvatures presents notable challenges. The low success rate observed in this study highlights the need for further refinement of the procedure to improve outcomes. Future research should investigate the Nd:YAP laser’s performance in a wider variety of anatomical scenarios, including narrow and curved canals, to fully evaluate its clinical utility. Also, it is important to mention that the irradiation parameters used in this study were suggested and based on our previous studies and are not part of preset parameters in a laser machine. Hence, because of the proven safety and relative efficacy, we advise the use of these irradiation parameters in further studies. Additional studies are also needed to explore alternative approaches that could enhance success rates in treating teeth with pronounced root curvatures.

## Conclusions

The proposed treatment approach for bypassing or extracting broken instruments proved to be less effective in cases involving teeth with pronounced root curvatures. The complexity of treating teeth with greater than 15 degrees of curvature presented significant challenges, which hindered the success of the Nd:YAP laser-assisted approach. While the protocol remains a safe and relatively fast method, it is clear that the Nd:YAP laser’s efficacy in managing fractured instruments in curved-rooted teeth needs further investigation.
